# A controllable gelatin-based microcarriers fabrication system for the whole procedures of MSCs amplification and tissue engineering

**DOI:** 10.1093/rb/rbad068

**Published:** 2023-08-14

**Authors:** Zixian Wang, Xiuxiu Zhang, Limin Xue, Gangwei Wang, Xinda Li, Jianwei Chen, Ruxiang Xu, Tao Xu

**Affiliations:** Precision Medicine and Healthcare Research Center, Tsinghua-Berkeley Shenzhen Institute (TBSI), Tsinghua University, Shenzhen 518055, People’s Republic of China; Precision Medicine and Healthcare Research Center, Tsinghua-Berkeley Shenzhen Institute (TBSI), Tsinghua University, Shenzhen 518055, People’s Republic of China; Department of Research and Development, Huaqing Zhimei (Shenzhen) Biotechnology Co., Ltd., Shenzhen 518107, People’s Republic of China; Department of Emergency, The Third Affiliated Hospital, Sun Yat-sen University, Guangzhou 510630, People’s Republic of China; Department of Neurosurgery, Sichuan Provincial People’s Hospital, University of Electronic Science and Technology of China, Chengdu 610072, People’s Republic of China; Bio-intelligent Manufacturing and Living Matter Bioprinting Center, Research Institute of Tsinghua University in Shenzhen, Tsinghua University, Shenzhen 518057, People’s Republic of China; Department of Neurosurgery, Sichuan Provincial People’s Hospital, University of Electronic Science and Technology of China, Chengdu 610072, People’s Republic of China; Precision Medicine and Healthcare Research Center, Tsinghua-Berkeley Shenzhen Institute (TBSI), Tsinghua University, Shenzhen 518055, People’s Republic of China; Department of Neurosurgery, Sichuan Provincial People’s Hospital, University of Electronic Science and Technology of China, Chengdu 610072, People’s Republic of China; Bio-intelligent Manufacturing and Living Matter Bioprinting Center, Research Institute of Tsinghua University in Shenzhen, Tsinghua University, Shenzhen 518057, People’s Republic of China

**Keywords:** microfluidics, size-controllability, gelatin-based microcarriers, MSCs expansion, tissue engineering

## Abstract

Biopolymer microbeads present substantial benefits for cell expansion, tissue engineering, and drug release applications. However, a fabrication system capable of producing homogeneous microspheres with high precision and controllability for cell proliferation, passaging, harvesting and downstream application is limited. Therefore, we developed a co-flow microfluidics-based system for the generation of uniform and size-controllable gelatin-based microcarriers (GMs) for mesenchymal stromal cells (MSCs) expansion and tissue engineering. Our evaluation of GMs revealed superior homogeneity and efficiency of cellular attachment, expansion and harvest, and MSCs expanded on GMs exhibited high viability while retaining differentiation multipotency. Optimization of passaging and harvesting protocols was achieved through the addition of blank GMs and treatment with collagenase, respectively. Furthermore, we demonstrated that MSC-loaded GMs were printable and could serve as building blocks for tissue regeneration scaffolds. These results suggested that our platform held promise for the fabrication of uniform GMs with downstream application of MSC culture, expansion and tissue engineering.

## Graphical Abstract

**Figure:**
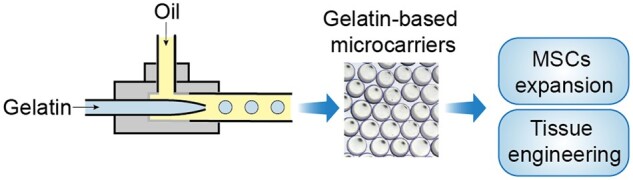

## Introduction

Microspheres have surfaced as a burgeoning technique for the efficacious delivery of cells and drugs [1–3], attributable to their high surface area-to-volume ratio that promotes cellular proliferation and expedites substance exchange. Significantly, microspheres have been utilized in the expansion of mesenchymal stromal cells (MSCs) [4–6] in response to the escalating demand for these cells in a multitude of applications such as cell therapies, tissue engineering and cellular factories producing secretory products [7–9]. The requisite dose of MSCs for each patient, for example, is estimated to be at least tens of millions and can even soar to several billion [10]. However, conventional planar culture methods were inadequate in meeting this demand due to their exorbitant material consumption and labor-intensive workload during passaging, monitoring and final harvest. Therefore, there was an urgent need for novel strategies for large-scale cell cultivation.

Contributing to a high surface area-to volume ratio, microspheres have been developed for scalable and efficient cellular production and evolved as microcarriers, first termed in 1960s [11]. Microcarriers, usually ranging from 100 to 300 μm, are defined as supportive matrices conducive to the culture and expansion of anchorage-dependent cells in the stirred bioreactors [12]. Commercially available microcarriers include dextran-based (Cytodex-1 and Cytodex-3), polystyrene-based (Hillex II-170, ProNectin F, FACT III), glass-based (Rapidcell, G2767), cellulose-based (Cytopore-2) and gelatin-based (Cultispher S, 3D TableTrix) [13], while gelatin-based microcarriers (GMs) exhibits superior proliferation and harvest properties [14, 15].

Despite advances in microcarrier technology, size uniformity remains a significant challenge, particularly for GMs. For example, the diameter of the Cultispher series varies from 130 to 380 μm [10], exceeding the recommended size distribution of within 25 μm [16], corresponding to a coefficient of variation (CV = standard deviation/mean) below 10%. This lack of homogeneity in size can result in uneven cell seeding distribution, ultimately impacting proliferation rates and final yields. Specifically, high seeding densities have been shown to impede karyokinesis, while low densities promote it [14]. Furthermore, batch-to-batch inconsistencies in yield may arise due to the nonuniformity of microcarriers.

Traditional microsphere preparation methods, such as spray drying, water-in-oil emulsion, and electrospray, have been unable to meet the requirements for uniformity [3, 17]. Therefore, several emerging approaches have been developed to optimize the uniformity of gelatin-based microspheres [15, 18, 19], as detailed in Supplementary Table S1. Electrohydrodynamic printed gelatin methacryloyl (GelMA) microspheres exhibited high roundness and relatively uniform size [18], but the numerous parameters of electro-assisted ejecting complicated the process and impeded the stability with a diameter ranging over 100 μm in some cases. Another study fabricated GelMA microspheres by a 3D digital light processing technology layer-by-layer [19], whereas the lowering roundness might cause uneven shear force in the bioreactors, which was harmful to the growing cells. Additionally, the variability of the diameter (11 ± 1 μm) relates to the layer height (set to 20 μm), and as the size of the microbeads decreased, the CV increased. Flow-focusing microfluidics presents another approach of uniform fabrication with the CV of the microspheres lower than 5% [15], but the fabrication of polydimethylsiloxane chips in the laboratory settings requires photoetching devices and processes with a high technical threshold. Moreover, dealing with the gel-clogged channels is intractable for the undetachable chips.

**Figure 1.**
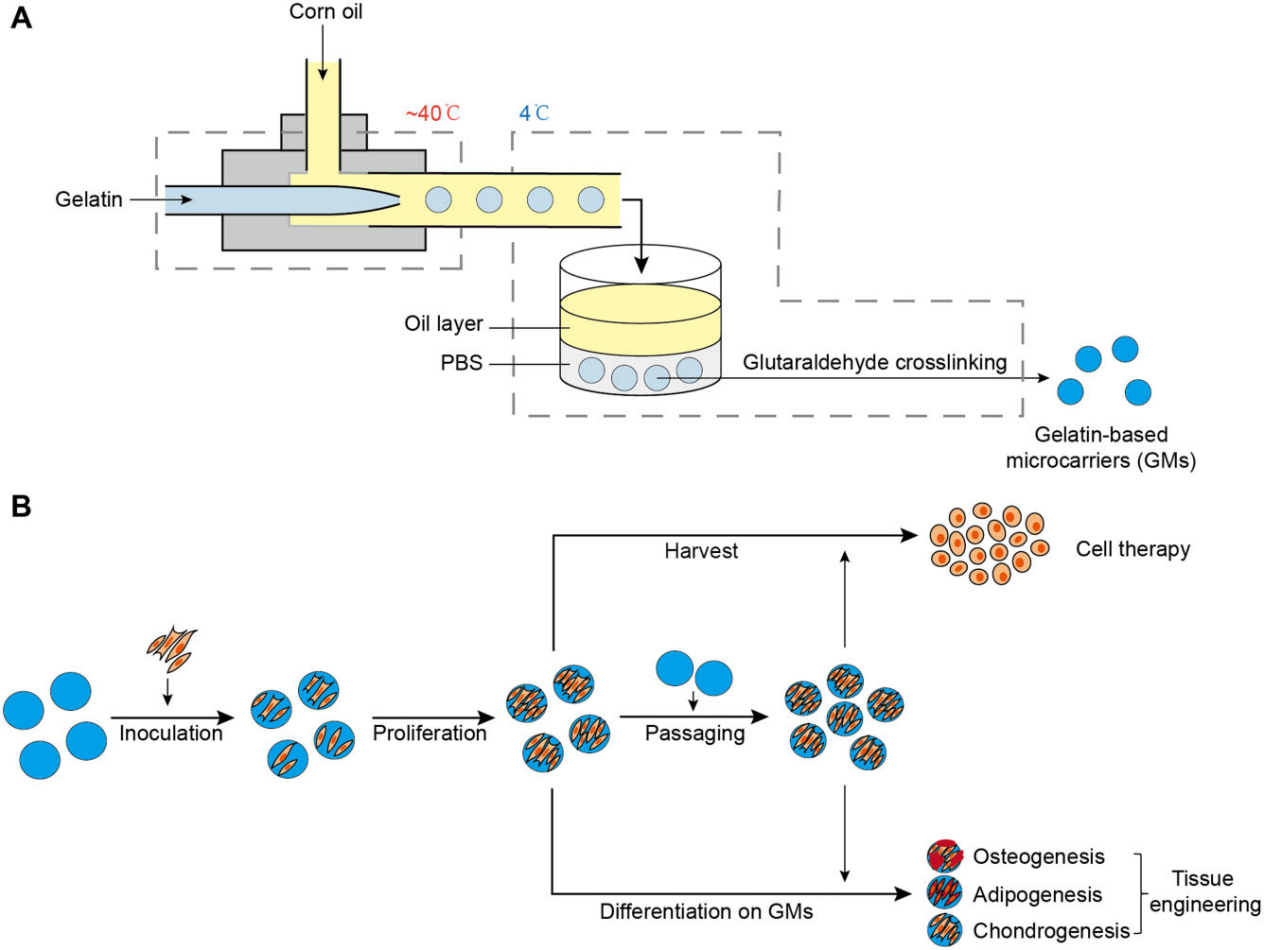
(A) Schematic illustration of fabrication of GMs by co-flow microfluidics. (B) Whole procedures of MSCs expansion and tissue engineering based on GMs.

Microfluidics enables the precise fabrication of microspheres [20], based on which we established a co-flow microfluidic system with lower cost by assembling glass capillaries together. The merits of this system include detachability, low cost, size-controllability and the ability to produce uniform microspheres with stability. Based on this system, uniform spherical GMs with size-turnability were fabricated and utilized in the whole procedures of MSCs amplification including adhesion, proliferation, passaging and harvesting, as well as extension for tissue engineering (Fig. 1).

## Materials and methods

### Assembling of the co-flow microfluidic chip

Three glass capillaries were integrated with an acrylic mold into the microfluidic chip: one for gelatin solution inlet with a tip diameter of 100 μm and two straight tubes for corn-oil inlet and outlet, respectively (Fig. 1A). These tubes were fixed by ultraviolet curing adhesive (Leaftop) and could be detached by heating up over 60°C.

### Fabrication of GMs

The microsphere fabrication system consisted of a microfluidic chip, two syringe pumps, a heater, an ice box and a collection dish. Gelatin (Aladdin) was dissolved in distilled water at 50°C to acquire a final concentration of 15%(w/v). The fully dissolved gelatin solution was loaded in a 1 ml syringe and maintained a liquid state by an infrared heater (Beurer). Corn-oil (Macklin) containing 1% Span 80 (Sigma-Aldrich) was loaded into a 50 ml syringe. The flow rate of the oil phase (the pump from Byond Medicine) was set to 1 ml/min, while the flow rate of gelatin solution (the pump from LongerPump) was optimized for stability and fabrication efficiency. Gelatin microbeads were collected in pre-cooled phosphate-buffered saline (PBS; Biochannel), overlaid with liquid paraffin (Macklin), and centrifuged at 600 rpm for 2 min. Then the microbeads were washed three times with cooled PBS to remove residual oil before being crosslinked with a 0.3% glutaraldehyde solution (Aladdin) at 4°C overnight. Excess aldehyde groups were quenched using a 0.2 M sodium borohydride (NaBH_4_) solution (Sigma-Aldrich) as a reducing agent [21]. Excess NaBH_4_ was then removed through sufficient washing with PBS until no air bubbles remained. Finally, the gelatin microbeads were sterilized by autoclaving prior to cell culture.

### Preparation of commercialized microcarriers

Cultispher S microcarriers (M9043; Sigma-Aldrich) were prepared in accordance with the manufacturer’s protocol. Briefly, the dry powder was weighed and dissolved in PBS before being sterilized by autoclaving. Subsequently, the microcarriers were washed twice with PBS and incubated in culture media for 2 h prior to inoculation.

### Characterization of the microcarriers

The diameter distribution and CV of GMs and other microcarriers were assessed using bright field microscopy (Nikon T2i). To visualize their microstructure, GMs were frozen at −80°C overnight and lyophilized for over 8 h. Gold nanoparticles were then applied as a coating for 120 s prior to scanning electron microscopy (SEM; PhenomScientific) observation. Pore sizes were quantified using ImageJ software (National Institutes of Health) based on SEM images.

### MSC culture

#### MSCs culture on the planar dish

Human adipose-derived mesenchymal stem cells (MSCs) were purchased from Sciencell and expanded on polystyrene petri dishes. The cells were cultured in a 37°C and 5% CO_2_ incubator (Memmert) using MSC culture media (MSCM; Sciencell) supplemented with 10% fetal bovine serum and 1% antibiotics. For passaging, the culture media was removed and replaced with 0.25% trypsin-EDTA (Gibco) for a 1-min incubation at 37°C. MSCs from passages 4 to 6 were used for subsequent experiments.

#### MSCs culture on microcarriers

Anti-attachment agent-treated 12-well plates (a component of SpheroX; from Engineering for Life [EFL]) were used to culture MSCs on microcarriers to minimize adhesion to the plate. A spinning mini-bioreactor with a rotation speed of 40–50 rpm was assembled according to the literature [22]. Each well contained approximately 3000 GMs (mean diameter of 380 μm) in 3 ml of culture media, providing a theoretical surface area of about 13.6 cm^2^. To roughly maintain a consistent surface area, nearly 10 000 Cultispher S particles (average diameter of 205 μm) were added to each well. Culture media was half-changed every 2 days, and the plates were incubated at 37°C and 5% CO_2_.

#### Seeding and harvest

Inoculation densities were designated as ‘Low’ (10 cells/GM), ‘Middle’ (50 cells/GM) or ‘High’ (100 cells/GM). Since there were approximately 3000 GMs in each well, the cell number for inoculation was 3 × 10^4^, 1.5 × 10^5^ and 3 × 10^5^, respectively. Attachment efficiency was estimated by counting unattached cell numbers in the supernatant at both 4 and 24 h post-inoculation. To minimize cell attachment to the bottom and walls of the well, an anti-attachment agent and a mini-spinning bioreactor were utilized as described in ‘MSCs culture on microcarriers’ section.

GelMA lysis buffer (EFL) contains a low concentration of collagenase, which has been used to dissolve gelatin microspheres and release cells [23]. Five days post-inoculation, the microspheres were harvested by applying a final concentration of 1 mg/ml GelMA lysis buffer and rotating the suspension in the bioreactor for 30 min to fully dissolve the microspheres. The cell suspension was then centrifuged at 1800 rpm for 4 min, and the supernatant was carefully aspirated. The cell sediment was subsequently resuspended in media containing trypan blue (0.04%; Solarbio). Cell numbers and viability were determined using a cell counting machine (Countstar). The following formula defined the expansion fold:


$$\mathrm{Expansion}\;\mathrm{fold}=\frac{\mathrm{Harvest}\;\mathrm{cell}\;\mathrm{number}}{\mathrm{Inoculation}\;\mathrm{cell}\;\mathrm{number}}$$

#### Addition of blank GMs and bead-to-bead transfer

Three days after seeding cells at a low inoculation density, an additional batch of 3000 blank GMs was introduced. Three days later, the harvesting procedure was performed.

### Characterization of MSC expanded on GMs

#### Live/dead staining

Live/dead staining was performed 24 h post-inoculation using calcein-AM and propidium iodide (PI) (both from Keygen Bio). Fluorescence images were captured using a Nikon Ti2 confocal microscope.

#### F-actin staining

F-actin staining was conducted to assess cell morphology using Actin-tracker Red-555 (diluted 1:100) and DAPI (both from Beyotime). Fluorescence images were captured using a Nikon Ti2 confocal microscope.

#### SA-β-gal staining

Approximately 2 × 10^4^ cells harvested from planar culture (P5 as negative control, P10 as positive control and P6 as non-treated group) or GMs expansion (replated at P6 as experiment group) were plated in 24-well plates and cultured for one days before staining with senescence-associated β-galactosidase (SA-β-gal) staining Kit (Beyotime), following the manufacturer’s instructions.

#### Flow cytometry

MSCs expanded on GMs were harvested and then stained for the positive markers, CD73-PE(BD Pharmingen), CD90-FITC(BioLegend), and CD105-BV421(BD Horizon), as well as negative markers CD34-PE and CD45-APC(both from BD Pharmingen). More than 1 × 10^4^ events for each group were acquired by CytoFLEX flow cytometer and analyzed with FlowJo software Ver. 10.6 (BD Biosciences).

#### Differentiation of MSC

For differentiation of adipose-derived MSCs, osteogenic (HUXMD-90021), adipogenic (HUXMD-90031), and chondrogenic (HUXMD-90041) differentiation and characterization kits from Oricell were utilized. MSCs from planar culture at passage 6, harvested from GMs or cultured on GMs were differentiated and characterized for osteogenic, adipogenic and chondrogenic lineages using respective culture and characterization kits according to the manufacturer’s instructions.

### Printability of GMs loaded with MSCs

Briefly, a 10% GelMA solution (in PBS) containing 0.3% lithium phenyl-2,4,6-trimethylbenzoyl phosphate (LAP) as photo-initiators was mixed with MSCs-laden GMs in a volume ratio of 1:2 at 37°C. The bead-suspended blend was transferred to a 1 ml syringe loaded on a LivPrint N bioprinter (Medprint) and used to fabricate a grid human-shaped model with a layer height of 0.4 mm. Extrusion speed was set to 0.09 mm/s while nozzle movement speed was set to 18 mm/s using a 20 G nozzle. The printing process was carried out at room temperature with ultraviolet light at 405 nm used for crosslinking. The scaffold was incubated in MSCM with media changes every 2 or 3 days. To assess the impact of the printing process on cell viability, live/dead staining was performed using calcein-AM and PI. Fluorescence images were captured using a Nikon Ti2 confocal microscope (capture depth 100 μm).

### Statistical analysis

All data were expressed as arithmetic mean ± standard deviation. Statistical analysis including analysis of variance and *t*-test was performed using GraphPad Prism Ver. 9.0. Differences were considered significant at *P* < 0.05 and marked in the figures; non-significant differences are not shown.

## Results

### Characteristics of GMs

**Figure 2.**
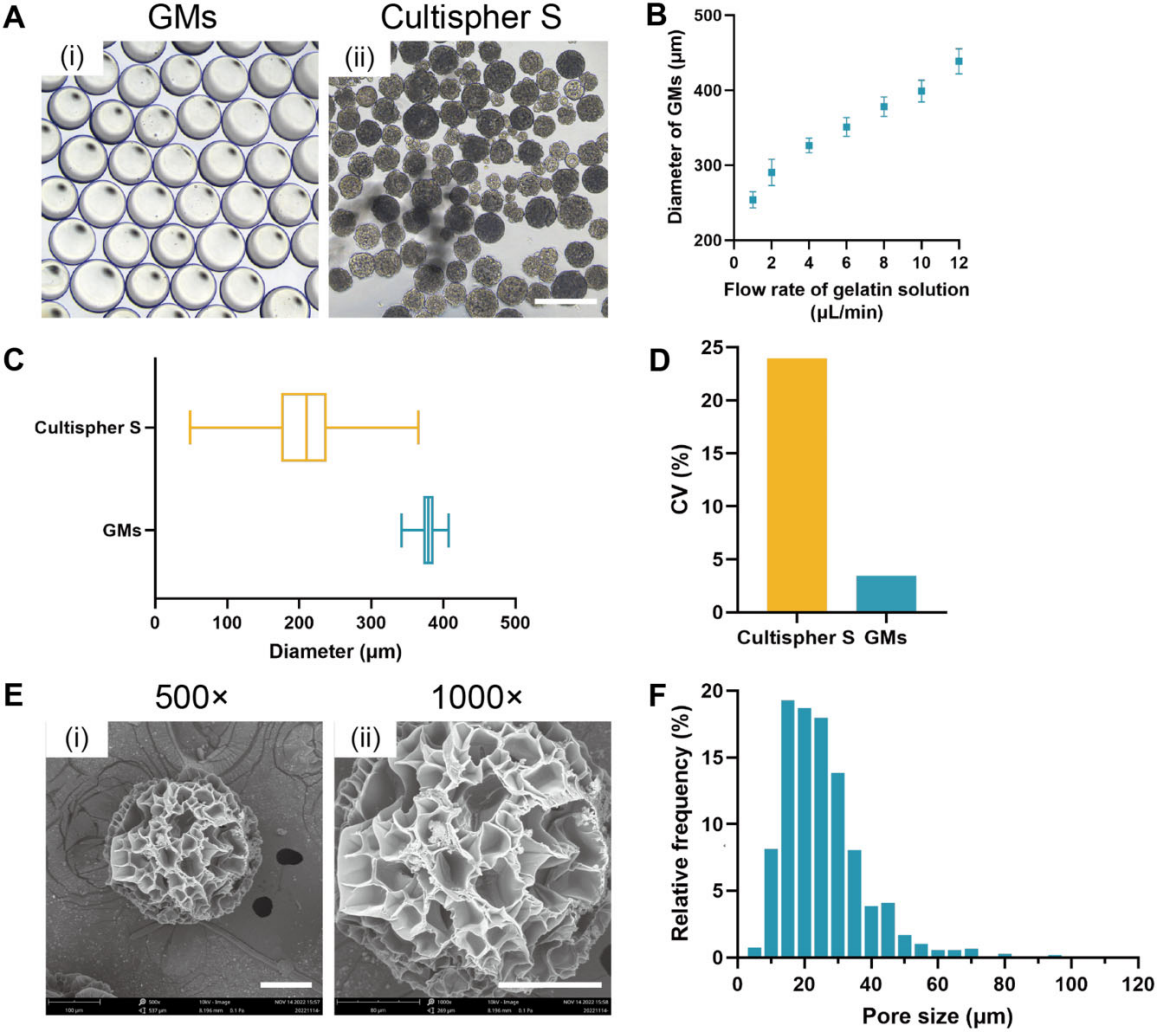
Characterization of the GMs. (A) Representative bright field images of (i) GMs and (ii) Cultispher S. Scale bar, 500 μm. (B) Diameter of GMs increased along with the increase of flow rate of gelatin solution (*n* = 120 for each flow rate). (C) Diameter distribution of Cultispher S and GMs, exhibiting average, upper and lower quartile, minimum and maximum (*n* = 270 for Cultispher S, *n* = 120 for GMs). (D) CV of diameter distribution of Cultispher S and GMs. (E) Representative SEM images of GMs, (i) ×500, (ii) ×1000. Scale bar, 100 μm. (F) Pore sizes distribution of GMs.

GMs fabricated using droplet microfluidics demonstrated precise size-tunability and exceptional uniformity. By modulating the flow rate of the gelatin solution, we were able to produce GMs with diameters spanning from approximately 250 to 450 μm (Fig. 2B). Theoretically, via adjustment of the tip diameter of the capillaries and the flow rate, a wider range of GMs could be achieved. To optimize manufacturing efficiency, we maintained a constant flow rate of the gelatin solution at 8 μl/min for subsequent experiments, yielding GMs with a diameter of 378 ± 13.0 μm. These GMs exhibited a narrow diameter distribution (CV = 3.4%) compared to Cultispher S, another gelatin commercial microcarriers (CV = 23.9%) (Fig. 2C and D). The wide range of diameter distribution (48.6–365.1 μm) observed in the commercial microcarrier could engender variability in cell yield from batch to batch [16]. Thus, for standardized expansion processes, uniform GMs are recommended.

SEM images of freeze-dried GMs revealed an abundance of micropores (average pore size: 25.6 ± 12.7 μm) (Fig. 2E and F), providing a greater surface area than predicted by theoretical calculations. The preponderance of pore sizes was distributed within the range of 10–50 μm. Notably, prior to lyophilization, GMs were spherical and exhibited high transparency; however, these characteristics were lost following freeze-drying (Supplementary Fig. S2).

### MSCs expansion on gelatin microcarriers

**Figure 3.**
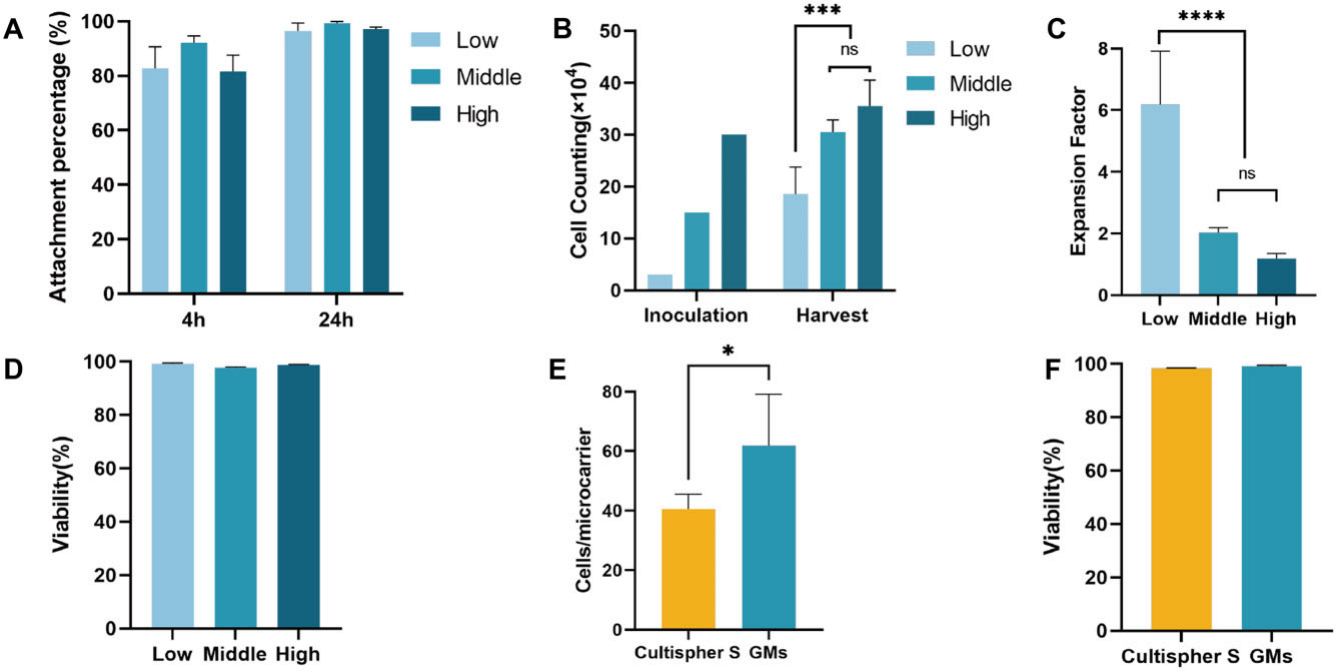
Attachment, expansion and harvest of MSCs on GMs. (A) Attachment efficiency of MSCs seeded on GMs at 4 and 24 h. (B) Cell numbers of inoculation and harvest at different seeding strategies. ***P < 0.001. (C) Expansion factor and (D) viability of MSCs cultured on GMs at different seeding densities. ****P < 0.0001. (E) Comparison of Cultispher S and GMs on cell number per microcarrier at ‘low’ seeding density. *P < 0.05. (F) Comparison of viability of Cultispher S and GMs at ‘low’ seeding density.

GMs produced by microfluidics could participate in the whole procedures of MSCs expansion and exhibit promising performance in cellular attachment and proliferation. Although the stirring bioreactor (40–50 rpm) impeded initial cell attachment efficiency, with only 78–90% of cells adhering to the GMs during 4 h of culture, this number reached 99% 1 day later (Fig. 3A). Live/dead staining 24 h post-inoculation indicated high cell viability and neglectable cytotoxic effect of glutaraldehyde residue on MSCs (Supplementary Fig. S1).

As for proliferation, three seeding densities were evaluated to determine optimal expansion efficiency: ‘Low’ (10 cells/GM), ‘Middle’ (50 cells/GM) and ‘High’ (100 cells/GM), corresponding to the inoculation of 3 × 10^4^, 1.5 × 10^5^ and 3 × 10^5^ cells, respectively (Fig. 3B). After 5 days of cultivation, the yield for ‘Low’ was 1.86 × 10^5^ cells (a 6.2-fold increase), for ‘Middle’ was 3.05 × 10^5^ cells (a 2.0-fold increase) and for ‘High’ was 3.55 × 10^5^ cells (1.2-fold) (Fig. 3B and C). The ‘Low’ seeding density significantly enhanced cell proliferation compared to the ‘Middle’ and ‘High’ densities (Fig. 3C), and all the inoculation strategies exhibited equally high viability (>98%) (Fig. 3D). The ‘Middle’ seeding strategy was still recommended for maximal yield within a limited period due to its relatively high baseline. In contrast, at ‘High’ density, karyokinesis was nearly static, indicating an upper limit for the load on each microsphere. In terms of load capacity comparison, each Cultispher S microcarrier could accommodate approximately 40 cells while our GMs could hold more than 60 cells each (Fig. 3E). Regardless of whether GMs or Cultispher S were used as microcarriers for MSCs culture and subsequent harvest via collagenase treatment, high cell viability was observed in both cases (Fig. 3F).

**Figure 4.**
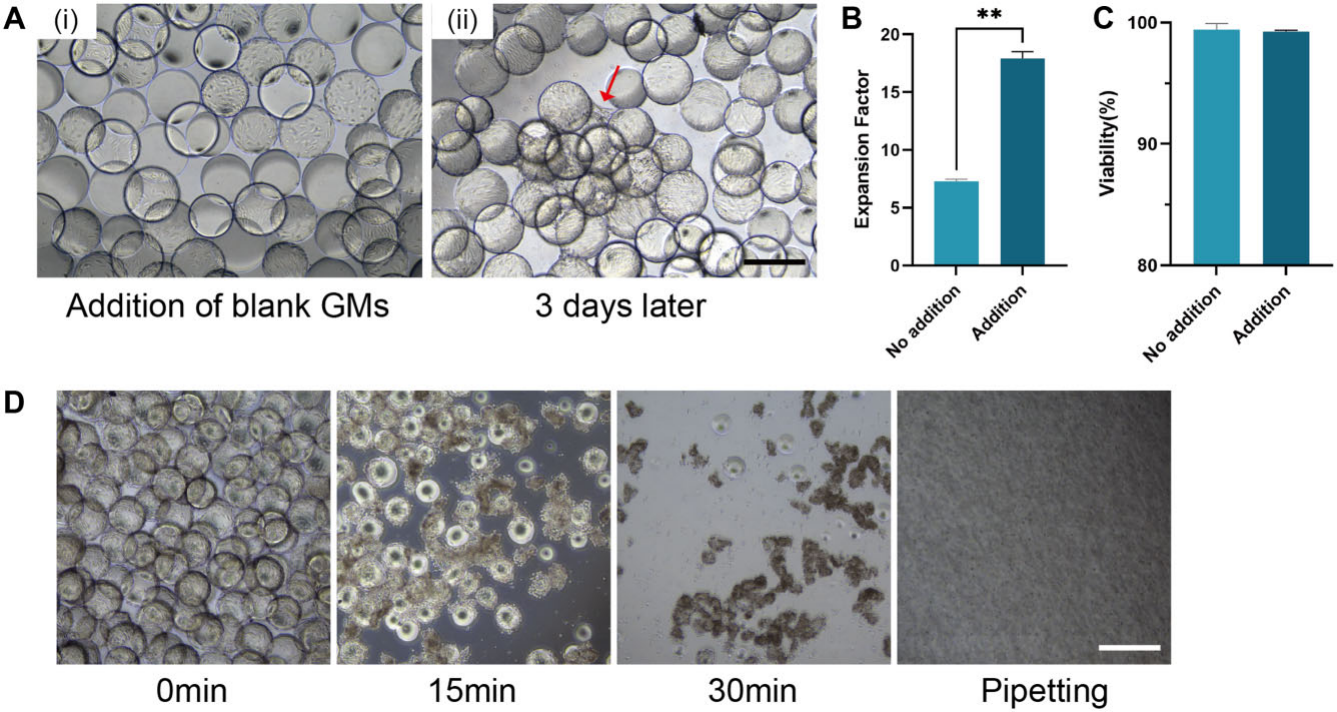
Passaging and harvesting based on GMs. (A) Bright field images of (i) addition of blank GMs on day 3, (ii) blank GMs covered by MSCs 3 days later due to cell migration. Red arrow indicates bead-to-bead transfer. Scale bar, 500 μm. (B) Expansion factors and (C) viability of MSCs at blank GMs addition strategy (passaging). **P < 0.01. (D) Time-elapse images of MSCs harvest procedure treated by collagenase. Scale bar, 500 μm.

The employment of GMs has engendered significant enhancements in both attachment and proliferation and also streamlined the passaging and harvest process. We evaluated the practicability of optimizing passaging via bead-to-bead transfer by introducing blank GMs 3 days post-inoculation to augment the growth area. Subsequent harvesting was executed another 3 days later and cell yield employing this GM addition stratagem increased 17-fold compared to the control group with no addition (7.5-fold), while preserving high viability (>98%) (Fig. 4B and C). Besides, the harvest process was simplified as treatment with collagenase. After digestion with GelMA lysate for 30 min, most GMs had degraded and MSCs were dispersed into single cells following pipetting (Fig. 4D).

### Characteristics of MSCs expanded on GMs

**Figure 5.**
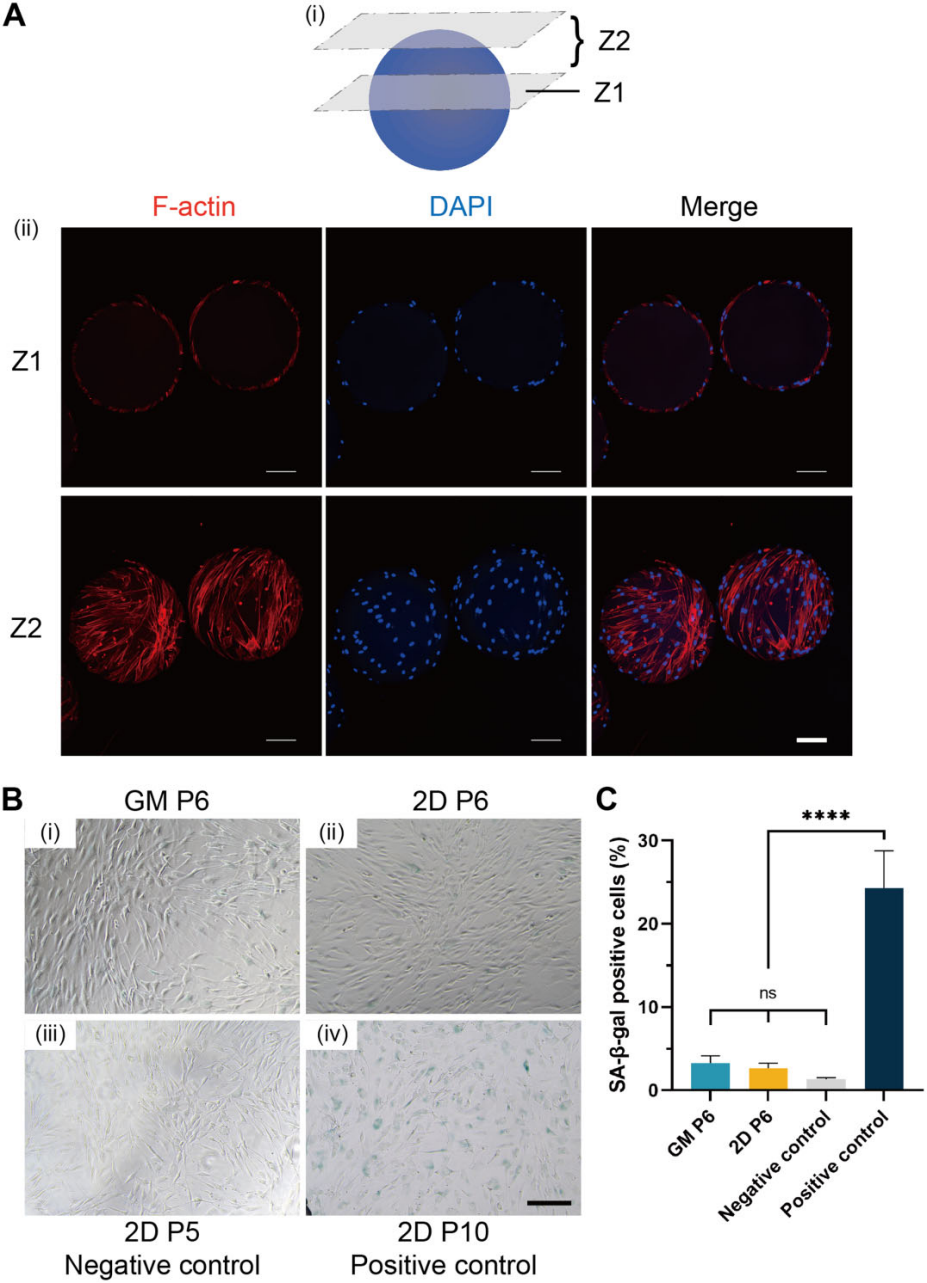
Morphology and senescence of MSCs cultured on GMs. (A) (i) Illustration of the pictured layers, Z1 for single layer and Z2 for overlap. (ii) Representative fluorescence images of MSCs growing on GMs, stained with phalloidin (F-actin-tracker, red) and DAPI (nucleus-tracker, blue). scale bar, 100 μm. (B) SA-β-gal staining of MSCs (i) on GMs (replated at P6), at (ii) 2D P6, (iii) 2D P5 as negative control, (iv) 2D P10 as positive control. Blue plaques meant SA-β-gal-positive staining. Scale bar, 200 μm. (C) Comparison of SA-β-gal-positive cells proportions of each group. ****P < 0.0001

Cell therapies necessitate equal consideration of both cell quantity and quality. Characterization of MSCs cultured on GMs encompassed general properties and MSC identifications. The former included the spreading and aging, while the latter contained the identification of surface markers and differentiation. The morphology of MSCs propagated on GMs was spindle-like, analogous to 2D culture, as evidenced by F-actin staining, with cells growing exclusively on the surface of non-porous GMs (Fig. 5A). Furthermore, SA-β-gal staining revealed no increase in β-galactosidase activity in MSCs expanded on GMs compared to 2D culture, indicating an absence of senescence in MSCs cultured on GMs (Fig. 5B and C).

**Figure 6.**
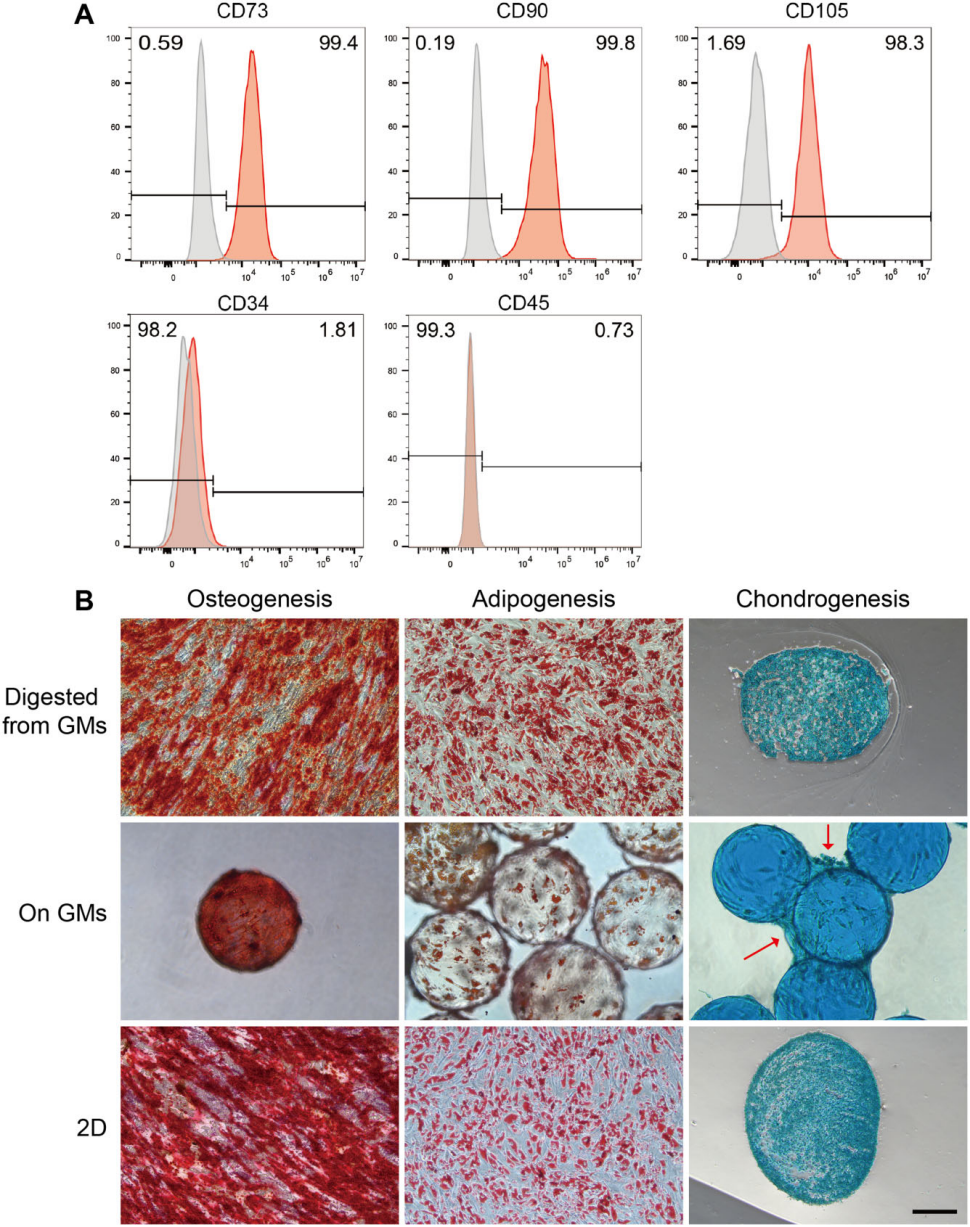
Surface markers and differentiation of MSCs expanded on GMs. (A) Flow cytometry analysis of surface markers of MSCs expanded on GMs. (B) Multipotency of MSCs expanded on GMs (digested from GMs or remaining on GMs) or planar culture as control. Alizarin red, oil red and alcian blue were used to stain with osteogenic, adipogenic and chondrogenic differentiation markers, respectively. Because GMs were stained with alcian blue, red arrows show cartilage-like components. Scale bar, 200 μm.

Cellular surface markers were identified via flow cytometry and MSCs harvested from GMs exhibited positive staining (>95%) for CD73, CD90 and CD105 along with negative staining (<2%) for CD34 and CD45 (Fig. 6A), consistent with established MSC identification criteria [24]. MSCs multipotency enables differentiation into osteoblasts, adipocytes and chondroblasts *in vitro*. Analogous to planar culture, MSCs harvested from GMs retained trilineage differentiation capacity and could be stained with osteogenesis, adipogenesis and chondrogenesis indicators providing potential for microbead-based tissue regeneration (Fig. 6B).

### GMs-based tissue engineering

**Figure 7.**
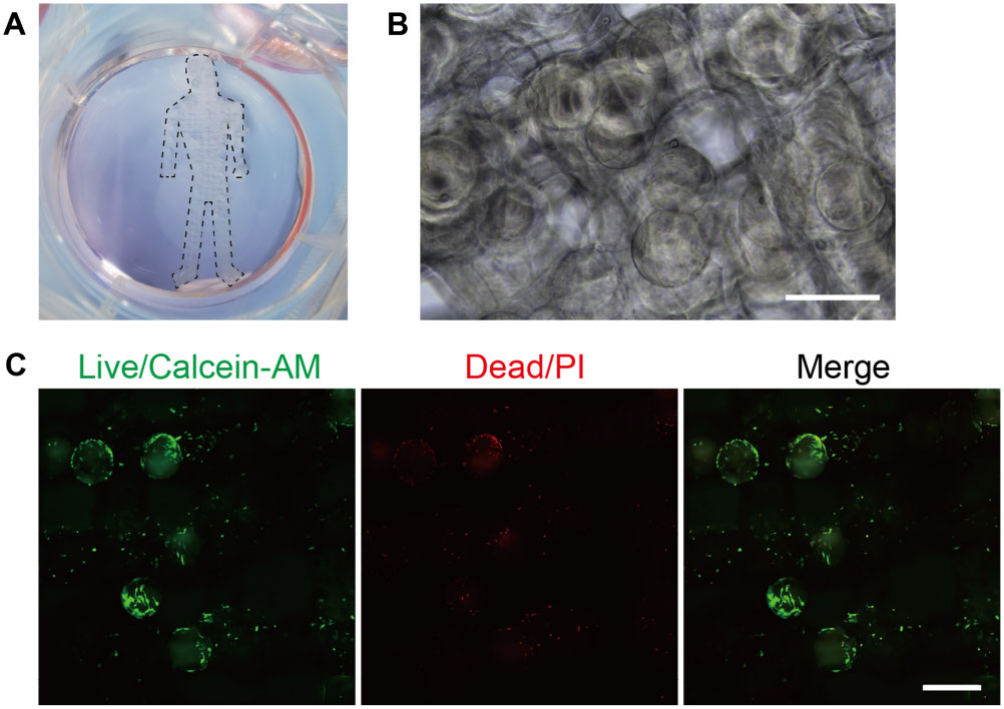
Tissue engineering based on MSCs-loaded GMs. (A) A human-shaped scaffold composed by MSCs-loaded GMs, marked with dotted lines. (B) Bright field images of the scaffold. Scale bar, 500 μm. (C) Live/dead staining with Calcein-AM and PI. Scale bar, 500 μm.

MSCs on GMs could be induced differentiated, and these biologically functional micro-tissue units hold promise for tissue regeneration when employed as cellular delivery vehicles to lesion sites. Furthermore, we appraised the printability of MSCs-loaded GMs for secondary fabrication into tissue engineering scaffolds. A grid-based structure in the shape of a human was constructed (Fig. 7A), with GMs interspersed within the filaments (Fig. 7B). To preclude aggregation and blockage of GMs, printing was executed on day 4 post-inoculation when the cells were not full confluent. Despite being subjected to mechanical extrusion and ultraviolet exposure during printing, the majority of MSCs on the GMs remained viable (Fig. 7C). By incorporating induced differentiation as previously delineated, the scaffolds could be transformed into osteal-like, adipose-like or chondral-like structures providing potential for tissue engineering applications predicated on microcarriers.

## Discussion

Droplet microfluidic devices provide precise control and high throughput for microsphere preparation [25, 26]. In this study, we presented a co-flow microfluidic platform for the fabrication of homogeneous and size-controllable GMs, with the advantages of detachability, stability and low cost. As a foundational technology, the GMs could have extensive applicability in the entire MSC amplification procedures including adhesion, proliferation, passaging and harvest, as well as tissue engineering. Due to the biocompatibility, dissolvability and uniformity, GMs enhanced the whole processes of MSCs culture with improved efficiency in cellular attachment, expansion and harvest, while optimizing passaging and harvest protocols.

### Exceptional cellular adhesion efficiency

Due to the presence of RGD (a tripeptide consisting of arginine, glycine and aspartate) motifs, gelatin inherently demonstrates superior performance in cell adhesion [27]. On the contrary, commercial microcarriers are typically composed of dextran (e.g. Cytodex-1 and Cytodex-3), polystyrene (e.g. Hillex II-170, ProNectin F, FACT III and CGEN 102-L) or glass (Rapidcell, G2767) and often require surface modification to enhance cell attachment and spreading [13]. A study by Schop et al. compared the seeding efficiency of dextran- and polystyrene-based microcarriers and found that Cytodex-1 outperformed its counterparts with an attachment efficiency of only 57% after 18 h [28]. The same study also reported that plastic substrates had the lowest attachment efficiency (approximately 35%), which could be improved through gelatin coating.

Cell attachment efficiency is also modulated by the agitation mode. In the short term (several hours), delayed (0 rpm) and intermittent agitation (a cycle of 60 rpm × 5 min and 0 rpm × 20 min) have been shown to be conducive to cell attachment, while no significant difference was observed between these modes and constant agitation (60 rpm) in 24 h of culture [14]. However, static incubation is not advised due to the non-uniform distribution of cells. In our study, we found that constant rotation at a rate of 40–50 rpm did not impede cell adhesion over an extended period; after 24 h of culture, nearly all cells (>99%) were anchored on GMs. These findings are consistent with those of Tristan et al., who reported that only 13% of cells adhered to collagen-coated microcarriers within 4 h under constant agitation but reached 100% attachment after 24 h [29]. Another study compared gelatin microcarriers with commercial microcarriers such as Cytodex series and SoloHill collagen and found equally high attachment efficiency (95–98%) under mild intermittent agitation (30 rpm× 2 min and 0 rpm × 28 min) [15], highlighting the future direction of intermittent agitation.

### Reliable cell expansion

The initial inoculation density of MSCs impacted subsequent multiplying since MSCs were sensitive to cellular density [30]. Generally, theoretical values of 3–5 cells per microcarrier were recommended [31], but the number was influenced by various parameters such as the microcarrier selection, cell type and concentration. In our study, ‘Low’ cell seeding density (10 cells/microcarrier), equivalent to 2200 cells/cm^2^, was employed since the GMs possessed a relatively large diameter. We observed the highest expansion factor (6.2-fold) after 5 days of culture compared to other seeding densities tested, indicating that the low density facilitated more rapid proliferation. Another study found that an ultra-low inoculation density (1111 cells/cm^2^) significantly increased the proliferation rate with an expansion factor of 29.8 ± 3.0 within 4 days; this superior outcome might also be related to the porous structure of the 3D TableTrix used in that study [14]. Therefore, in practical manufacturing applications using microcarriers, optimization of seeding strategies is necessary to maximize cell yield.

Collagen derivatives have been explored for microcarriers like Cultispher series, but Cultispher G was found to exhibit a lower proliferation rate compared to both Cytodex series and polystyrene-based microcarriers [32]. Nowadays, 3D TableTrix microcarriers made from gelatin have attracted attention due to their exceptional expansion efficiency (over 500-fold increase in 11 days) [14], demonstrating the potential for gelatin to serve as an excellent substrate for microcarriers. We compared our GMs with Cultispher S, another microcarrier based on gelatin, under identical culture conditions to minimize interference from material components. Our GMs exhibited a higher cell accommodation capacity than Cultispher S; however, this difference was attributed to variations in diameter. Notably, Cultispher S features a porous structure with an increased surface area (Supplementary Fig. S3). To generate porous GMs, we employed a freeze-drying strategy and observed preserving micropores even after overnight rehydration. Resultantly, the expansion factor of freeze-dried GMs rose to 15 with high viability (95%) (Supplementary Fig. S2). Distinct nano-scale patterns were observed in the porous structures generated by different methods and parameters. For example, freeze-drying produced non-connected micro-holes while the use of porogens such as hydrocarbonates resulted in contiguous cavities [33]. Although the porous structure enhanced surface area and cellular proliferation, it also reduced transparency and hindered immediate observation of cell confluence via microscopy due to pore interference with light beams.

### Protocols optimization of passaging and harvesting

Typical planar passaging operations involve a complex series of steps including media removal, washing with PBS, treating with trypsin-EDTA solution, trypsin quenching with serum, centrifugation, supernatant aspiration and cell resuspension in media prior to replating. This labor-intensive process requires significant reagent consumption in large-scale production and increases the risk of contamination. However, microcarrier technology can greatly simplify passaging through bead-to-bead transfer [34], where the cells migrated from one microsphere to another upon the addition of new microcarriers. Furthermore, cell proliferation rates increase with the addition of new batches of microspheres due to reduced cell density promoting cell division. Despite the advantages of microcarrier-based culture, it is necessary to specifically define the passage number and the impact of bead-to-bead transfer by characterizing cellular behaviors.

GMs facilitated workload reduction and enhanced harvest efficiency owing to their enzymatic degradability. In contrast, for microcarriers composed of indissolvable polymers such as dextran and polystyrene, harvest programs typically involve detaching cells from the microcarriers and separating the microcarriers from the cell suspension. The former step often involves treatment with trypsin for more than 12 min [35] while the latter step often employs filtration methods. However, achieving sufficient harvest using these methods can be challenging and may limit the final yield. For example, dissolvable microcarriers (from Corning) achieved harvest efficiency of 92 ± 4%, while the number of traditional filtration-based methods was only 45 ± 3% [36]. Another microcarrier composed of alginate/chitosan could be degraded by trypsin/EDTA solution and exhibited higher detachment efficiency (55%) than Cytodex-1 (38%) [37]. Another study reported over 90% harvest efficiency using 0.1% Pronase (from *Streptomyces griseus*) solution without agitation for 5 min [15]. On the contrary, Cytodex and SoloHill collagen microcarriers exhibited harvest efficiencies of approximately 60% and 70%, respectively. However, the viability of the cells was not evaluated in that work. Our results demonstrated that over 98% of MSCs remained viable following 30-min treatment with collagenase indicating its mildness. In our study we employed GelMA lysate (crude collagenase extract) to dissolve the microcarriers, but the precise type and concentration of collagenase utilized was not ascertained.

### Tissue engineering potential

Tissue engineering scaffolds with microbeads have been investigated for the regeneration of various tissues including bone, cartilage, skin, heart, liver and nerve [38]. These composites can be classified as microsphere-incorporating and microsphere-based depending on scaffold composition. The former contains additional supportive materials, while the latter utilizes solely microspheres as building blocks. Advantages of scaffolds with cell-loaded microcarriers include improved control of cellular delivery, enhanced mechanical properties and compartmentalized biofunctionalized units [39]. For instance, Xu et al. built the composite structure by adding the microbeads into a supportive poly(ε-caprolactone) (PCL) scaffold for cartilage tissue regeneration [40]. However, this structure lacked integration and controllable arrangement of the microbeads. Here, we employed extrusion printing for scaffold construction, and a human-shaped scaffold was built with GMs distribution in the filaments. As previously demonstrated, MSCs on the microcarriers maintained multipotency and could differentiate into corresponding cell types upon treatment with induced differentiation media. Nevertheless, this represents merely an incipient step toward the fabrication of microcarrier-incorporating scaffolds and further research is required to optimize bio-ink composition and print parameters to enhance viability and functionalization.

### Preparation efficiency of GMs by co-flow microfluidics

The production efficiency of our droplet microfluidics system could be enhanced, as the current flow rate of gelatin is only 8 μm/min, yielding approximately 15 000 particles per hour. One potential approach to increase efficiency is to adjust the flow rates of the dispersed and continuous phases. While increasing the flow of either phase can shorten the droplet formation interval [41], this method has a finite potential for efficiency improvement. An alternative approach is to integrate multiple droplet generators. Nisisako and Torii demonstrated the scalability of co-flow microfluidics by developing a microfluidic module with 128 co-flow geometry units, achieving a throughput of 2.13 ml/min [42]. Thus, for the mass production of gelatin microspheres, designing an array of multiple droplet generators may be a more efficient approach.

## Conclusion

We demonstrated co-flow microfluidics for the fabrication of size-controllable and homogeneous GMs and implemented them into MSCs expansion and 3D bioprinting. Gelatin serving as the raw material of microcarriers exhibited superior biocompatibility and enhanced efficiency in the whole procedures of MSCs culture, including inoculation, proliferation, passaging, harvesting and tissue engineering. High attachment efficiency, proliferation rate and harvest efficiency, combined with workflow simplification of passaging and harvesting, contributed to increasing yield and reduced cost, facilitating scalability. Furthermore, we proved the injectability of MSCs-laden GMs, as the composition of tissue regeneration scaffolds. In conclusion, co-flow microfluidics served as a platform for the manufacture of uniform microspheres, and these microspheres based on gelatin could serve as potential habitats for MSCs and other anchorage-dependent cell types to realize scalable culture and production, as well as tissue engineering.

## Supplementary Material

rbad068_Supplementary_Data
