# Supplementary material for: A controllable gelatin-based microcarriers fabrication system for the whole procedures of MSCs amplification and tissue engineering
Source: Regen Biomater. 2023 Aug 14;10:rbad068. doi: 10.1093/rb/rbad068 (PMC10458456; doi:10.1093/rb/rbad068)
Supplement: rbad068_Supplementary_Data [file rbad068_supplementary_data.docx]

Supplementary materials

**A controllable gelatin-based microcarriers fabrication system for the whole procedures of MSCs amplification and tissue engineering**

Zixian Wang, Xiuxiu Zhang, Limin Xue, Gangwei Wang, Xinda Li, Jianwei Chen*, Ruxiang Xu*, Tao Xu*

Table of contents

Fig. S1. Fluorescence images of live/dead staining 24 h after inoculation

Fig. S2. Bright field images of freeze-drying GMs

Fig. S3. SEM images of Cultispher S

Table S1. Representative GMs preparation efficiency by different methods


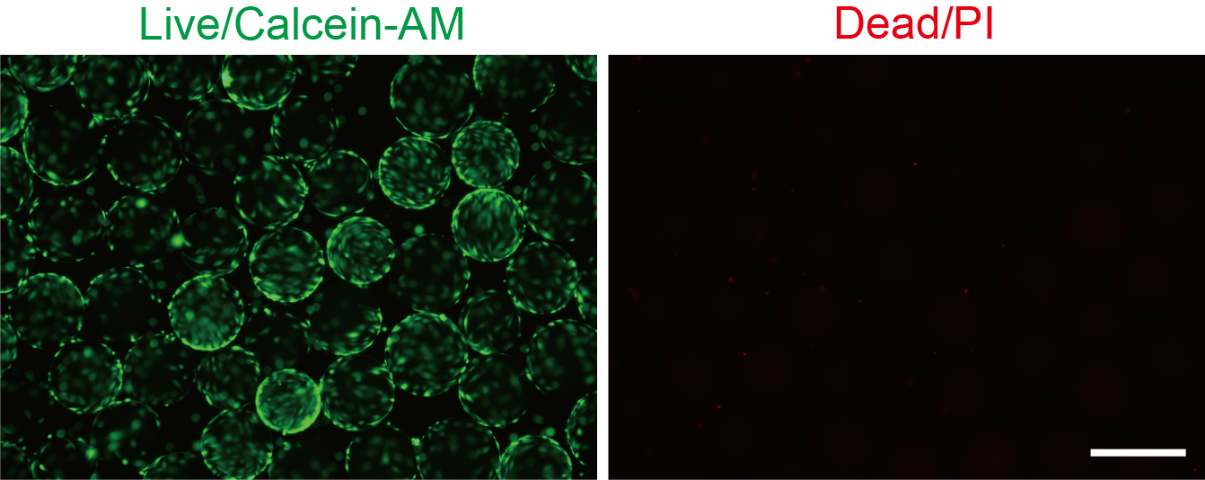


Figure S1. Representative fluorescence images of live/dead staining with Calcein-AM (green) and PI (red) at “High” seeding density 24 h after inoculation. Scale bar, 500μm.


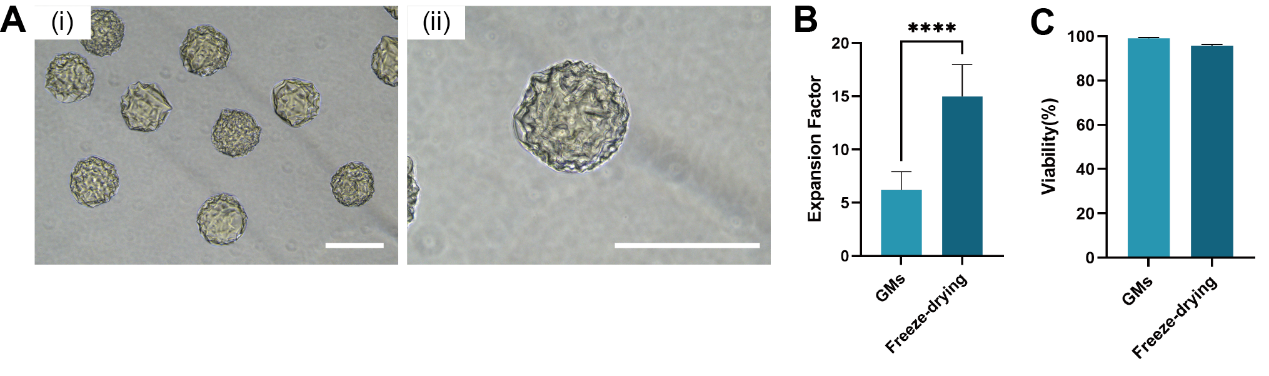


Figure S2. A) Bright field images of freeze-drying GMs at different magnification (i)40×, (ii)100×. Scale bar, 500 μm. B) Expansion factors and C) Viability of MSCs on freeze-dried GMs. *****P*<0.0001.


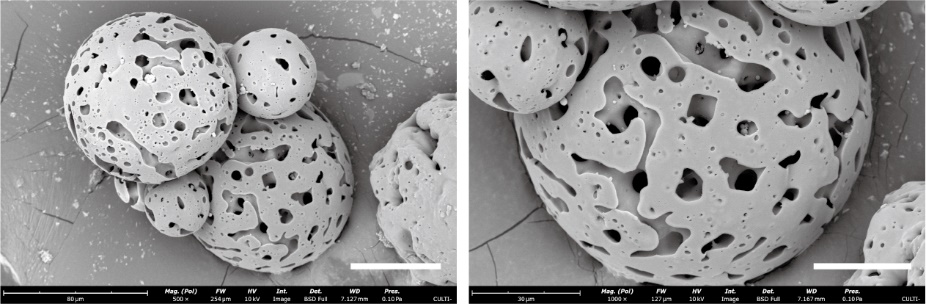


Figure S3. Representative SEM images of Cultispher S with rich micropores. (i)500×, Scale bar, 50 μm. (ii)1000×, Scale bar, 25 μm.

**Table S1.** Representative GMs preparation efficiency by different methods

| Methods | Preparation efficiency (mL/h) | Diameter (mean ± standard deviation) (μm) | Reference |
| --- | --- | --- | --- |
| Water-in-oil emulsion | 11 | 169.3±41.3 | [1] |
| Spray drying | 360 | 15.7±4.2 | [2] |
| Electrospray | 3 | 217.7±57.4 | [3] |
| Digital light processing | 660 thousand particles/hour | 203.82±10.13 | [4] |
| Microfluidics | 0.15 | Not indicated, CV<5% | [5] |

References:

[1] Contessi Negrini N, Lipreri M V, Tanzi M C, Farè S. In vitro cell delivery by gelatin microspheres prepared in water-in-oil emulsion. *J Mater Sci Mater Med* 2020;31:26.

[2] Qu S, Dai C, Yang F, Huang T, Xu T, Zhao L, Li Y, Hao Z. A Comparison of Two Methods for the Preparation Cefquinome-Loaded Gelatin Microspheres for Lung Targeting. *Pharm Res* 2018;35:43.

[3] Chen M, Li J, Shu G, Shen L, Qiao E, Zhang N, Fang S, Chen X, Zhao Z, Tu J, Song J, Du Y, Ji J. Homogenous multifunctional microspheres induce ferroptosis to promote the anti-hepatocarcinoma effect of chemoembolization. *J Nanobiotechnol* 2022;20:179.

[4] He Q, Liao Y, Zhang J, Yao X, Zhou W, Hong Y, Ouyang H. "All-in-One" Gel System for Whole Procedure of Stem-Cell Amplification and Tissue Engineering. *Small* 2020;16:e1906539.

[5] Ng EX, Wang M, Neo SH, Tee CA, Chen CH, Van Vliet KJ. Dissolvable Gelatin-Based Microcarriers Generated through Droplet Microfluidics for Expansion and Culture of Mesenchymal Stromal Cells. *J Biotechnol* 2021;16:e2000048.
